# Secondary structure models of 18S and 28S rRNAs of the true bugs based on complete rDNA sequences of *Eurydema maracandica* Oshanin, 1871 (Heteroptera, Pentatomidae)

**DOI:** 10.3897/zookeys.319.4178

**Published:** 2013-07-30

**Authors:** Shasha Yu, Yanhui Wang, Dávid Rédei, Qiang Xie, Wenjun Bu

**Affiliations:** 1 Institute of Entomology, College of Life Sciences, Nankai University, 94 Weijin Road, Tianjin 300071, China

**Keywords:** rRNA, secondary structure, Heteroptera, molecular synapomorphy, *Eurydema maracandica*

## Abstract

The sequences of 18S and 28S rDNAs have been used as molecular markers to resolve phylogenetic relationships of Heteroptera for two decades. The complete sequences of 18S rDNAs have been used in many studies, while in most studies only partial sequences of 28S rDNAs have been used due to technical difficulties of amplifying the complete lengths. In this study, we amplified the complete 18S and 28S rDNA sequences of *Eurydema maracandica* Oshanin, 1871, and reconstructed the secondary structure models of the corresponding rRNAs. In addition, and more importantly, all of the length variable regions of 18S rRNA were compared among 37 families of Heteroptera based on 140 sequences, and the D3 region of 28S rRNA was compared among 51 families based on 84 sequences. It was found that 8 length variable regions could potentially serve as molecular synapomorphies for some monophyletic groups. Therefore discoveries of more molecular synapomorphies for specific clades can be anticipated from amplification of complete 18S and 28S rDNAs of more representatives of Heteroptera.

## Introduction

Each cluster of rDNA in turn contains external transcribed spacer (ETC), 18S rDNA, internal transcribed spacer 1 (ITS1), 5.8S rDNA, ITS2 and 28S rDNA (Hillis and Dixon 1991). These genes and spacer regions, separately or in combination, have been used as molecular markers for resolving phylogenetic relationships of Heteroptera for two decades from Wheeler et al. (1993). Among them, the length-conservative regions of 18S rDNA and 28S rDNA have been mostly used for resolving phylogenetic relationships between higher level group (Wheeler et al. 1993, Xie et al. 2005, Shuh et al. 2009), while the length-variable regions (LVRs), ITS1 and ITS2 have been used at species level (Marcilla et al. 2001, Hebsgaard et al. 2004, Damgaard et al. 2005, Calleros et al. 2010).

As far as the rDNAs are concerned, complete sequences of 18S rDNA have been used in various phylogenetic studies on Heteroptera (Tian et al. 2008, Jung and Lee 2011). Comparatively, so far only partial sequences of 28S rDNAs have been involved in phylogenetic studies on Heteroptera due to technical difficulties of amplifying the complete lengths. These difficulties are twofold. First, the amplification of 28S rDNA is more likely interfered by the hairpin structures, or tandem replicates of single nucleotides or oligonucleotides. Second, universal primers are unavailable for amplifying the 28S rDNA of Heteroptera.

Reconstruction of the complete secondary structure model of 28S rRNA can only be achieved by full sequencing of 28S rDNA. According to the results of comparative studies of 18S rRNAs of Insecta (Xie et al. 2009) and Hemiptera (Xie et al. 2008), the secondary structure model can have significance in two aspects. First, the alignment results of rDNAs can be corrected based on the secondary structure models of the corresponding rRNAs (Kjer et al. 2006). Further, it is possible to evaluate the potential influences to the results of phylogenetic reconstruction. Second, the secondary structure model provides a novel view and datasets to discover synapomorphies, such as group-specific insertions or deletions (indels), exactly the same length of some LVRs, and length expansions. In this study, the secondary structure models of 18S and 28S rRNAs of Heteroptera were reconstructed based on corresponding rDNA sequences of the pentatomid species *Eurydema maracandica* Oshanin, 1871. Additionally, all LVRs of complete 18S rRNA and the Divergent region 3 (D3) of 28S rRNA were compared based on data of nearly all available families of Heteroptera from GenBank (www.ncbi.nlm.nih.gov/Genbank), with exception of a few problematic sequences.

## Methods

### Taxon sampling

According to the currently accepted classification of Heteroptera (Schuh and Slater 1995, Schuh et al. 2008), heteropterans are divided into 7 infraorders, 23 superfamilies and 76 families. In this study, the 18S rDNA dataset comprises 140 sequences representing 7 infraorders, 16 superfamilies and 37 families of Heteroptera. The 28S rDNA dataset, which only included the D3 region, comprises 84 sequences of 7 infraorders, 21 superfamilies and 51 families.

### Molecular experiments

The specimen was collected from Yining, Xinjiang Uyghur Autonomous Region, China (43°56'N, 81°19'E, 570m) on July 26, 2011 by Qiang Xie. In the alcohol-kept insects’ collection of Nankai University, the ID number for the voucher specimen is NKU0150177. The species was identified based on the available literature and comparative material, its identity was confirmed by P. Kment. The specimen perfectly fits the original description (Oshanin 1871) and the available detailed redescriptions and illustrations (Stichel 1961, Putshkov 1965, Zheng 1983, Nonnaizab 1988) of *Eurydema maracandica*.

Genomic DNA was extracted from thoracic tissue of ethanol-preserved specimen. Total genomic DNA was isolated using the CTAB-based method (Reineke et al. 2002). The primers Ns1 and Ns8 used for amplifying the 18S rDNA were those used by Barker et al. (2003). The others were designed by the software Primer Premier 5.00 (Lalitha 2000). These primer sets were used to amplify six overlapping fragments of 28S rDNA and two overlapping fragments of 18S rDNA. All of the sequences were sequenced in both directions. In addition, 18S rDNA was cloned, which used pEASY-T3 (TransGen, Beijing, China) as vector, following the manufacturer’s instructions. Amplification was carried out in a 50 µL volume reaction, with 1.5 units of *LA Taq* DNA Polymerase, 2.5 mM of dNTP and 10 µM of each primer. The thermal cycling program of PCR consisted of an initial denaturation at 94°C for 1 min, followed by 35 cycles (94°C for 30 s, 49–57°C for 30 s, 72°C for 1 min), and ending with a final extension at 72°C for 8 min. Colony PCR was carried out in a 25 µL volume reaction, and consisted of 30 amplification cycles. All of the primer pairs and annealing temperatures are listed in Supplementary File 1. The GenBank accession numbers of 18S and 28S rDNAs of *Eurydema maracandica* are JX997807 and JX997806 respectively.

### Reconstruction of secondary structure and Sequence alignment

The secondary structure of 18S rRNA was slightly revised from the universal model of insects (Xie et al. 2009) based on the sequence of *Drosophila melanogaster* (GenBank accession number M21017). In the group specific optimization of the universal model according to the sequences of Heteroptera, secondary structures of LVRs were reconstructed using the thermodynamics folding method of the software RNAstructure 5.4 (Reuter and Mathews 2010). The secondary structure of 28S rRNA was revised from the model of insects (Wang et al. 2013) based on the sequence of *Drosophila melanogaster* (GenBank accession number M21017). The numbering system for LVRs of 28S rRNA followed the D system (Hassouna et al. 1984, Ellis et al. 1986). In the optimization of the previous model of insects, secondary structures of LVRs were reconstructed using RNAstructure 5.4 as well. The re-calculated results in this study were selected under the principle of co-variation: the fewer the secondary structural elements, especially the paired regions, are destroyed by each sequence, the better the model is (Gutell et al. 2002). We simplified the principle in this study as: the longer the stems are kept by each sequence, the better the model is. Sequence alignment was performed by ClustalW, which is imbedded within BioEdit 7.1.3 (Hall 2011). Manual alignments were based on the rRNA secondary structure models reconstructed in this study.

## Data resources

The data underpinning the analyses reported in this paper are deposited in the Dryad Data Repository at doi: 10.5061/dryad.j8kp5

## Results and discussion

### General description of secondary structures

**Figure 1.**
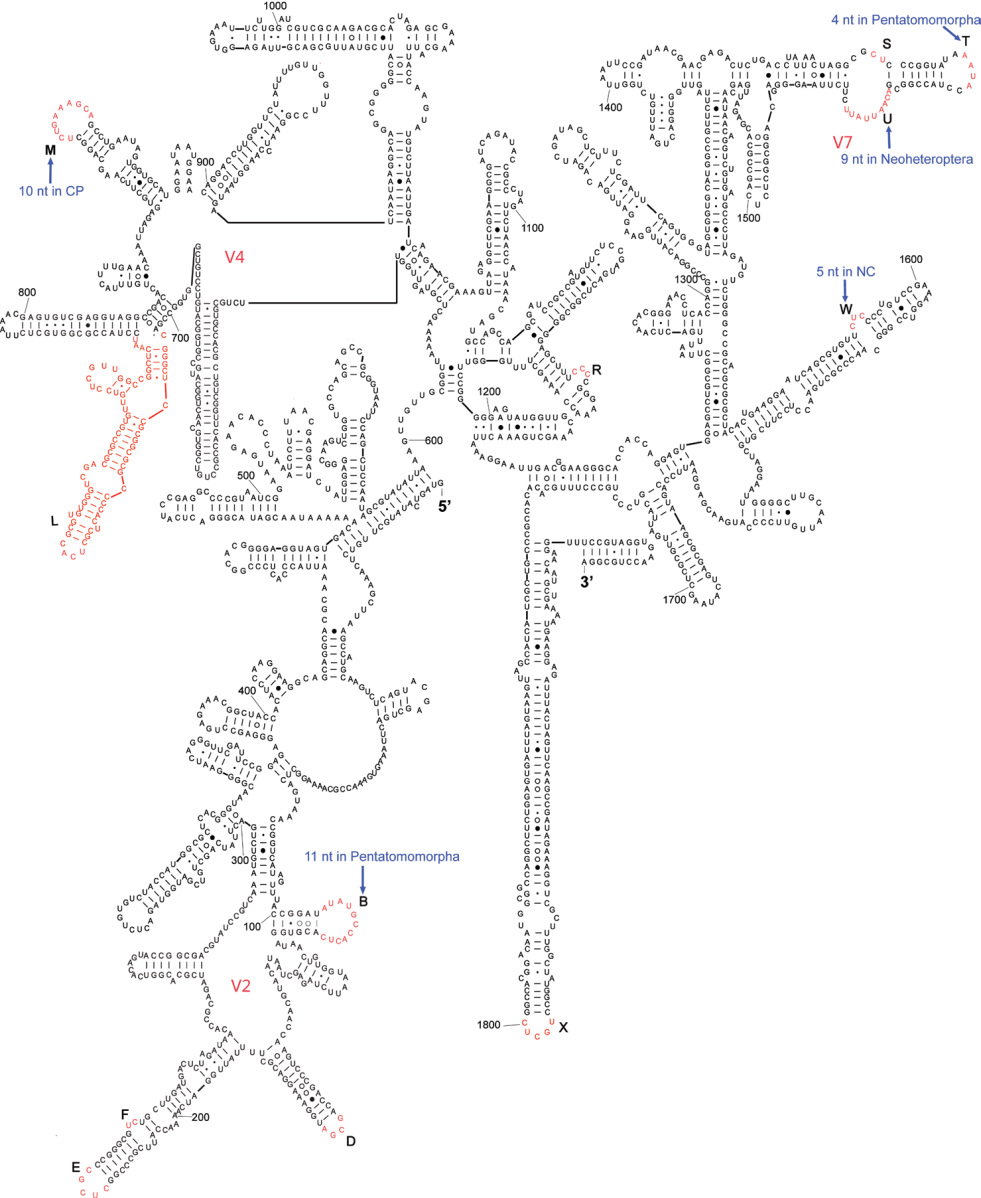
Secondary structure model of 18S rRNA of *Eurydema maracandica*. The bases marked in black represent length-conservative regions, and the bases labeled as capital letters **B** to **W** in red represent 13 LVRs. CP and NC represent monophyletic groups Cimicomorpha+Pentatomomorpha and Naboidea+Cimicoidea, respectively. Base pairing is indicated as follows: standard canonical pairs by lines (G–C, A–U), wobble G:U pairs by dots (G·U), A:G or A:C pairs by open circles (A○G, A○C), and other non-canonical pairs by filled circles (e.g., A●A).

The complete sequence of 18S rDNA of *Eurydema maracandica* is 1,894 bp. In the secondary structure of the corresponding rRNA (Fig. 1) the LVRs L and X regions were specifically optimized. The 18S rDNA sequence of *Eurydema maracandica* was aligned with other 140 orthologous sequences of 37 families in Heteroptera. The alignment result suggested that the local length variations of 18S rDNA are localized in 13 independent LVRs except for various indels. Most LVRs were restrained in three domains, which were previously named as V2, V4 and V7 (Neefs et al. 1993). Among all of the 13 LVRs, LVR L is the most variable one in length.

**Figure 2.**
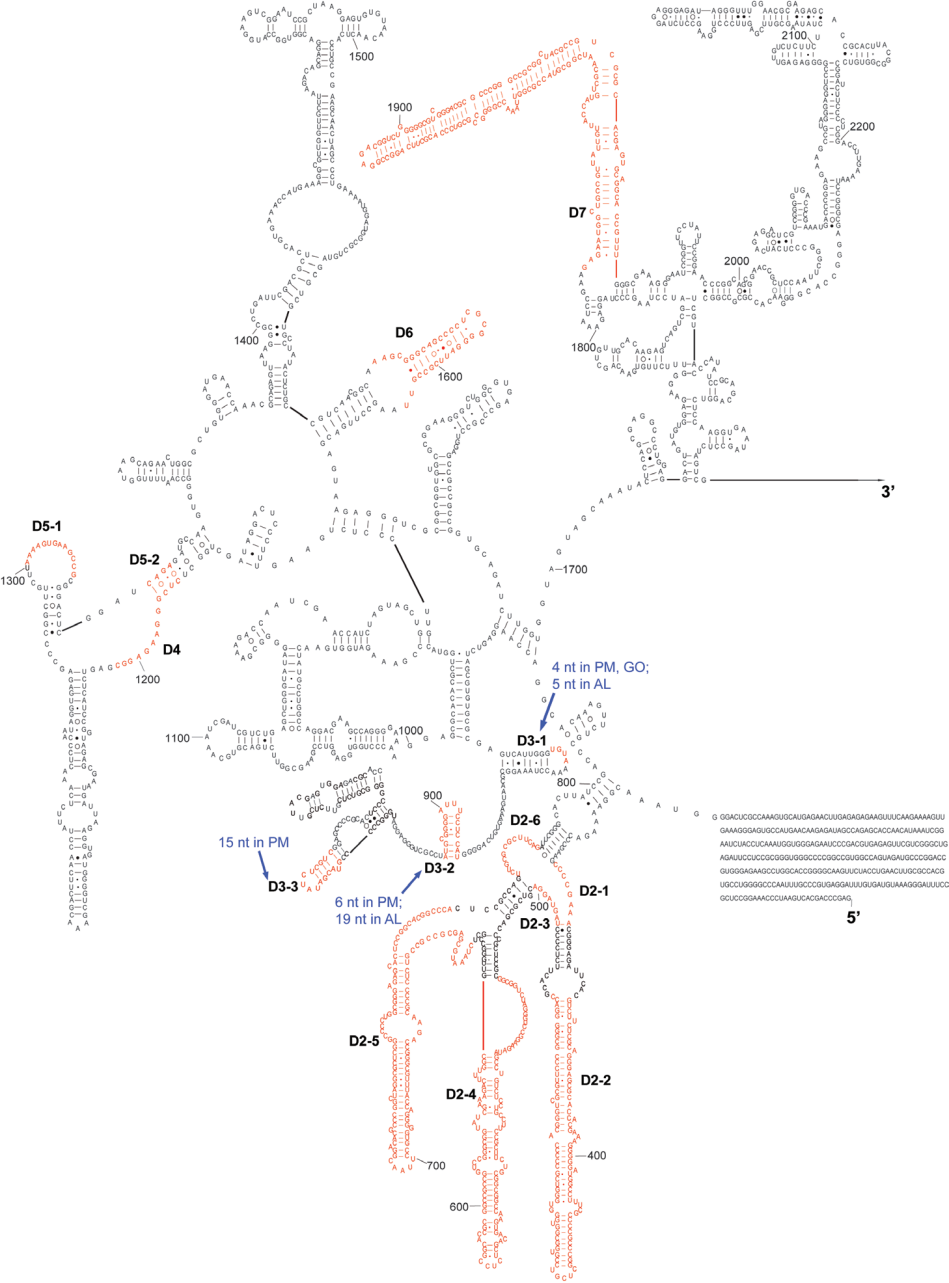
The 5’-half part of secondary structure model of 28S rRNA of *Eurydema maracandica*. The numbers D2 to D7 represent six LVRs. PM, GO and AL represent monophyletic groups Paraphrynoveliidae+Macroveliidae, Gelastocoridae+Ochteridae and Acanthosomatidae+Lestoniidae, respectively.

**Figure 3.**
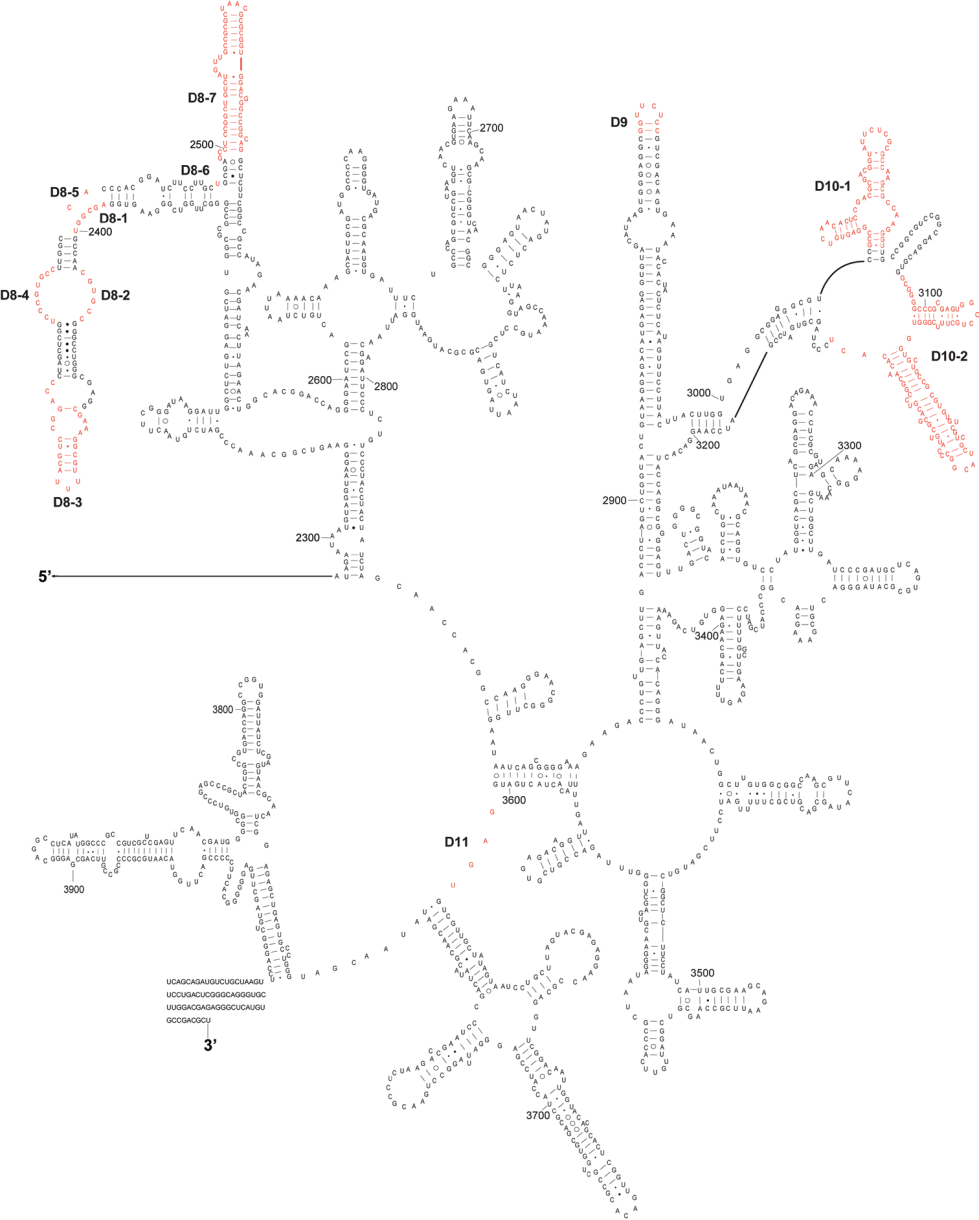
The 3’-half part of secondary structure model of 28S rRNA of *Eurydema maracandica*. The numbers D8 to D11 represent four LVRs.

The complete sequence of 28S rDNA of *Eurydema maracandica* is 4,030 bp. In the secondary structure of corresponding rRNA (Figs 2, 3), the LVRs D2, D3, D7, D8, D10, and D11 were specifically optimized. Compared with the secondary structure of the universal model of insect 28S rRNA, LVRs of heteropteran 28S rRNAs are distributed in 10 regions (D2–D11). Only D3 region of most superfamilies has corresponding data in GenBank, therefore only this region was comparatively analyzed and the results may serve as an inspiring case for the other LVRs of 28S rRNA. Alignment results suggested that in the D3 region, LVRs can be further divided into three sections (D3-1, D3-2 and D3-3), which are interspaced by several short length-conservative regions. Among these three separate LVRs, D3-2 is the most variable one in length.

According to the above results, the local length variations of heteropteran rRNAs are moderate in comparison with the length variations of eukaryotic rRNAs (Xie et al. 2011). The local length variations of heteropteran rRNAs did not cause overlap between adjacent regions (See Supplementary Files 2 and 3) like it was documented in the suborder Sternorrhyncha of Hemiptera (Xie et al. 2008) and some other insect orders (Xie et al. 2009). However, the ambiguity in alignment caused by length variations might have impact on the results of phylogenetic reconstruction of Heteroptera, and it deserves further evaluation.

### The LVRs potentially serving as synapomorphies

**Figure 4.**
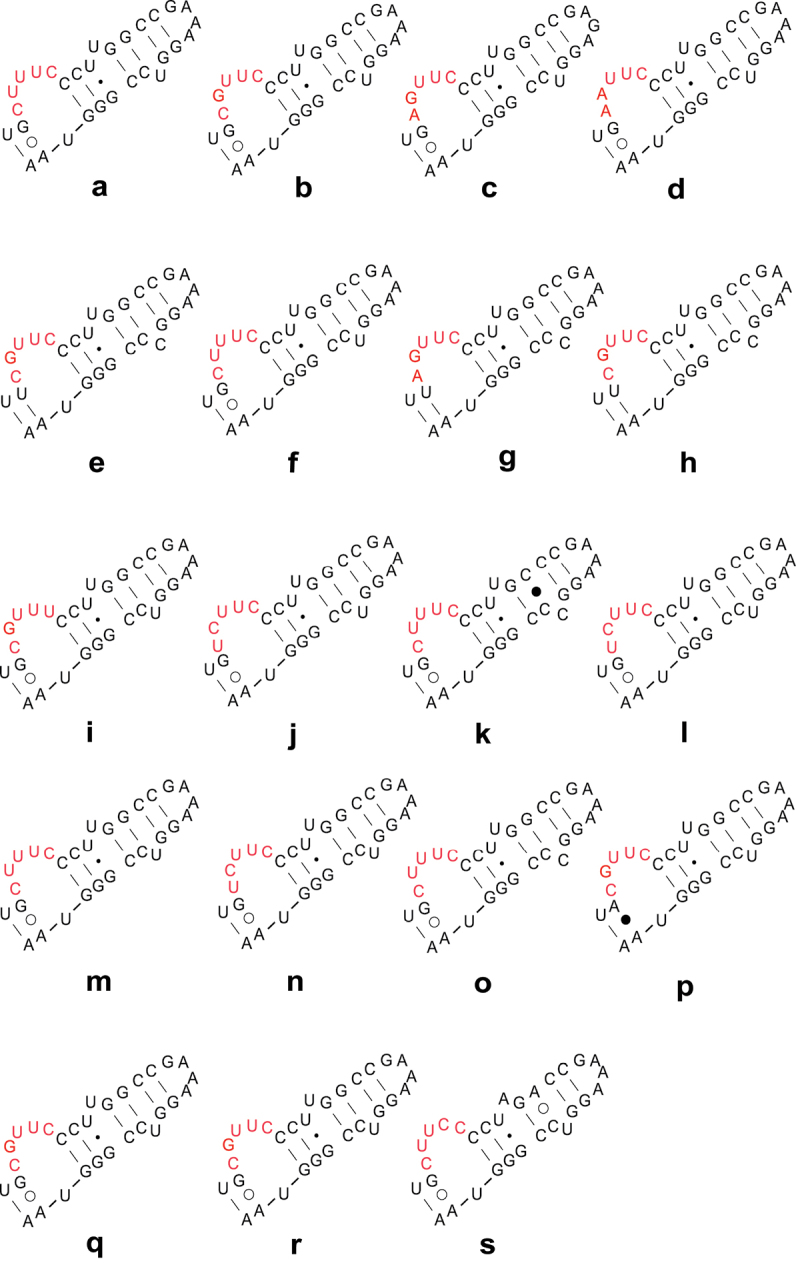
Secondary structure models of LVR W of Naboidea and Cimicoidea. These sequences are from 15 genera, 19 species of Naboidea and Cimicoidea. The species names and GenBank Accession numbers are as follow: Nabidae (**a**) *Nabis ferus* EF487300 (**b**) *Nabis flavomarginatus* GQ258424 (**c**) *Himacerus apterus* GQ258425; Lyctocoridae (**d**) *Lyctocoris beneficus* EF487298; Anthocoridae (**e**) *Anthocoris* sp. AY252319 (**f**) *Anthocoris confusus* EF487297 (**g**) *Anthocoris montanus* EF487307 (**h**) *Tetraphleps aterrimus* EF487295 (**i**) *Amphiareus obscuriceps* EF487301 (**j**) *Orius agilis* EF487296 (**k**) *Physopleurella armata* EF487308 (**l**) *Montandoniola moraguesi* EF487310 (**m**) *Xylocoris cerealis* GQ258395 (**n**) *Bilia* sp. GQ258406 (**o**) *Buchananiella crassicornis* GQ258407 (**p**) *Lasiochilus japonicus* GQ258410 (**q**) *Lasiochilus luceonotatus* GQ258411; Cimicidae (**r**) *Cimex lectularius* GQ258396; Curaliidae (**s**) *Curalium cronini* EU683128.

Campbell et al. (1994) hypothesized that length expansion in 18S rDNA could serve as synapomorphy for Sternorrhyncha. Later, the concepts of “molecular morpho-metrics” (Billoud et al. 2000) and “morpho-molecular structures” (Ouvrard et al. 2000) were introduced. It was found that length variation and indels could serve as synapomorphies for specific monophyletic groups of insects (Xie et al. 2009). The results of this study indicate that, the LVRs B, M, T, U and W of 18S rDNA, and D3-1, D3-2 and D3-3 of 28S rDNA could potentially serve as molecular synapomorphies (Table 1). According to the lengths of LVRs B and T of 18S rDNA, there only a few exceptions exist within Pentatomomorpha. Therefore, the 11nt length of LVR B and the 4nt length of LVR T very likely originate from the last ancestor of Pentatomomorpha and are the synapomorphies of this infraorder. In the same sense, the 10nt length of LVR M is a dominant state in the clade (Cimicomorpha+Pentatomomorpha), and the 9nt length of LVR U is very conservative within Neoheteroptera. Fig. 4 shows the secondary structure models of LVR W of Naboidea and Cimicoidea. Within Heteroptera, Naboidea and Cimicoidea have the same unique length of 5nt in this region. Taking the problem of the phylogeny within the superfamily Pentatomoidea for more instances, the 5nt length of LVR D3-1 and 19nt length of LVR D3-2 in 28S rRNA could possibly serve as synapomorphies for an Acanthosomatidae+Lestoniidae clade (Grazia et al. 2008). Additionally, the Paraphrynoveliidae+Macroveliidae clade shares the same length of LVR D3-1, D3-2 and D3-3. Gelastocoridae and Ochteridae have the same length of 4nt in D3-1 region. Therefore amplification of complete 18S and 28S rDNAs of more representatives very likely will result in more representative dataset for reconstruction of ancestral states and discoveries of more molecular synapomorphies for specific clades within Heteroptera.

**Table 1.** Monophyletic groups within Heteroptera with some LVRs serving as potential synapomorphies.

**Monophyletic group**	**Reference**	**LVR (number of sequences examined)**	
		**18S rDNA**	**28S rDNA**
Neoheteroptera	Štys et al. (1975)	9nt U (131)	
	Schuh (1979)		
	Wheeler et al. (1993)		
Paraphrynoveliidae+Macroveliidae	Damgaard (2008)		4nt D3-1 (3)
			6nt D3-2 (3)
			15nt D3-3 (3)
Gelastocoridae+Ochteridae	Hebsgaard (2004)		4nt D3-1 (4)
Cimicomorpha+Pentatomomorpha	Štys et al. (1975)	10nt M (117)	
	Schuh (1979)		
	Schaefer (1993)		
	Wheeler et al. (1993)		
Naboidea+Cimicoidea	Schuh et al. (2009)	5nt W (36)	
Pentatomomorpha	Leston et al. (1954)		
	Schuh (1979)	11nt B (39)	
	Schaefer (1993)	4nt T (37)	
	Wheeler et al. (1993)		
Acanthosomatidae+Lestoniidae	Grazia et al. (2008)		5nt D3-1 (8)
			19nt D3-2 (8)

Note: Detailed information see Supplementary Files 4 and 5.

## Appendix 1

Primer pairs and the annealing temperatures for amplifying rDNAs of *Eurydema maracandica*. (doi: 10.3897/zookeys.319.4178.app1) File format: Mircrosoft Word Document (doc).

**Explanation note:** List of primer pairs and annealing temperatures.

## Appendix 2

Alignment of 18S rDNA sequence of Heteroptera. (doi: 10.3897/zookeys.319.4178.app2) File format: DNA and Protein Sequence Alignment (fas).

**Explanation note:** The alignment of *Eurydema maracandica* 18S rDNA sequence with other 140 orthologous sequences of Heteroptera.

## Appendix 3

Alignment of D3 region in 28S rDNA sequence of Heteroptera. (doi: 10.3897/zookeys.319.4178.app3) File format: DNA and Protein Sequence Alignment (fas).

**Explanation note:** The alignment of *Eurydema maracandica* D3 region in 28S rDNA sequence with other 84 orthologous sequences of Heteroptera.

## Appendix 4

The summarization of length variation in 18S rDNA sequence of Heteroptera. (doi: 10.3897/zookeys.319.4178.app4) File format: Microsoft Excel Worksheed (xls).

**Explanation note:** The length of 12 LVRs in all currently known 18S rDNA sequences of Heteroptera.

## Appendix 5

The summarization of length variation in D3 region of 28S rDNA sequence of Heteroptera. (doi: 10.3897/zookeys.319.4178.app5) File format: Microsoft Excel Worksheed (xls).

**Explanation note:** The length of 3 LVRs in all currently known sequences of D3 region of 28S rDNA of Heteroptera.
