# Supplementary material for: Secondary structure models of 18S and 28S rRNAs of the true bugs based on complete rDNA sequences of Eurydema maracandica Oshanin, 1871 (Heteroptera, Pentatomidae)
Source: Zookeys. 2013 Jul 30;(319):363–77. doi: 10.3897/zookeys.319.4178 (PMC3764533; doi:10.3897/zookeys.319.4178)
Supplement: Supplementary file 1 — Primer pairs and the annealing temperatures for amplifying rDNAs of Eurydema maracandica. (doi: 10.3897/zookeys.319.4178.app1) File format: Mircrosoft Word Document (doc). [file ZooKeys-319-363-s001.doc]

**Supplementary file 1. Primer pairs and the annealing temperatures for amplifying rDNAs of *Eurydema maracandica***

| Gene | Primer pairs (5’→3’) | | Annealing  temperature |
| --- | --- | --- | --- |
| 18S rDNA | Ns1  18SP3 | GTAGTCATATGCTTGTCTC  GGTTAGAACTAGGGCGGTATCT | 49℃ |
|  | 18SP5  Ns8 | CAAGAACGAAAGTTAGAGGT  TCCGCAGGTTCACCTACGGA | 49℃ |
| 28S rDNA | SS7  XS2 | GCGGAGGAAAAGAAACTAAC  GGCATAGTTCACCATCTTTCG | 50℃ |
|  | DF1  FD1 | ATCCGACCCGTCTTGAAACAC  TGCTACTACCACCAAGATCTG | 55℃ |
|  | DE1  DG | GCTGTGGGATGAACCAAACG  GTTGGCACCGCTCCACTTCC | 57℃ |
|  | op  pob | TAGGAGGGAGATAGGGTTTG  TACCGCCCCAGTCAAACTCC | 55℃ |
|  | uz  zu | AGGTGTAGCATAAGTGGGAG  TTCGGTCTTAGAGGCGTTCAG | 55℃ |
|  | cb4  kb4 | CGGCTCTTCCTATCATTGCG  TGCCCGAGTCAGAAGTCGTCTAC | 53℃ |
